# 5D Flow Tensor MRI to Efficiently Map Reynolds Stresses of Aortic Blood Flow In-Vivo

**DOI:** 10.1038/s41598-019-55353-x

**Published:** 2019-12-11

**Authors:** Jonas Walheim, Hannes Dillinger, Alexander Gotschy, Sebastian Kozerke

**Affiliations:** 1 Institute for Biomedical Engineering, University and ETH Zurich, Zurich, Switzerland; 2 Department of Cardiology, University Hospital Zurich, Zurich, Switzerland

**Keywords:** Valvular disease, Biomedical engineering

## Abstract

Diseased heart valves perturb normal blood flow with a range of hemodynamic and pathologic consequences. In order to better stratify patients with heart valve disease, a comprehensive characterization of blood flow including turbulent contributions is desired. In this work we present a framework to efficiently quantify velocities and Reynolds stresses in the aorta *in-vivo*. Using a highly undersampled 5D Flow MRI acquisition scheme with locally low-rank image reconstruction, multipoint flow tensor encoding in short and predictable scan times becomes feasible (here, 10 minutes), enabling incorporation of the protocol into clinical workflows. Based on computer simulations, a 19-point 5D Flow Tensor MRI encoding approach is proposed. It is demonstrated that, for *in-vivo* resolution and signal-to-noise ratios, sufficient accuracy and precision of velocity and turbulent shear stress quantification is achievable. *In-vivo* proof of concept is demonstrated on patients with a bio-prosthetic heart valve and healthy controls. Results demonstrate that aortic turbulent shear stresses and turbulent kinetic energy are elevated in the patients compared to the healthy subjects. Based on these data, it is concluded that 5D Flow Tensor MRI holds promise to provide comprehensive flow assessment in patients with heart valve diseases.

## Introduction

Imaging is playing an increasing role in assessing the hemodynamic and structural consequences of aortic valve diseases^1^. Time-resolved volumetric mapping of blood flow velocities using 4D Flow MRI^2^ offers insights into changes of mean and peak velocities^3^, flow displacement^4^, vorticity and helicity^5^, wall shear rates^6^ and relative pressure gradients^7^. Besides the assessment of time-resolved velocity vector fields, the intensity of stochastic velocity fluctuations as encountered in transient and turbulent flows can be probed^8^.

In general, turbulence dissipates energy and increases resistance to flow, generating additional load for the cardiovascular system^9^. Moreover, the effective coefficient of friction in turbulent flows is higher compared to normal flow and hence shear forces acting on the formed elements in blood are accentuated, potentially leading to blood cell damage^10–12^.

It has been shown that by quantifying Turbulent Kinetic Energy (TKE), i.e. the energy stored in velocity fluctuations, important additional information is obtained relative to current clinical information in heart valve patients^13^. Beyond quantifying TKE, all components of the Reynolds stress tensor (RST) may be obtained using appropriate changes of the MRI pulse sequence design^14^, a concept that has been validated using simulation and simplified *in-vitro* experiments recently^15,16^. Such an approach may offer improved mapping of pressure gradients across heart valves and stenotic vessel sections^15–17^.

A key practical challenge to quantifying the RST *in-vivo* relates to the extended scan times required in order to encode velocity fluctuations along the minimum number of six non-collinear axes. In addition, the dynamic range of velocity fluctuations encountered *in-vivo* demands at least two measurements along each non-collinear axis^18^, leading to scan times well beyond clinically acceptable limits.

The objective of the present work was to develop an approach to efficiently map the RST and hence turbulent shear stresses *in-vivo* within clinically acceptable scan times. Our approach is based upon recent advances in compressed sensing and sparse recovery of respiratory-motion resolved 4D Flow MRI data, which we have presented previously^19^. Here we propose a framework to efficiently quantify velocities and the RST using a highly undersampled acquisition scheme with locally low-rank image reconstruction^20,21^ and multipoint encoding per axis including Bayesian estimation of average velocity per voxel as well as intravoxel velocity standard deviations^18^. We term this approach 5D Flow Tensor MRI.

Using a total of 19 velocity encodings, 5D Flow Tensor MRI requires 10 min of scan time and hence enables data acquisition in a clinical setting. To demonstrate accuracy and precision of 5D Flow Tensor MRI, results of computer simulations based on previously collected *in-vivo* data and *in-vitro* particle tracking velocimetry of valvular flow are shown. *In-vivo* proof of concept of 5D Flow Tensor MRI is demonstrated on patients with a bio-prosthetic heart valve revealing elevated turbulent shear stresses and turbulent kinetic energy compared to healthy controls.

## Results

### MRI data acquisition and reconstruction

**Figure 1:**
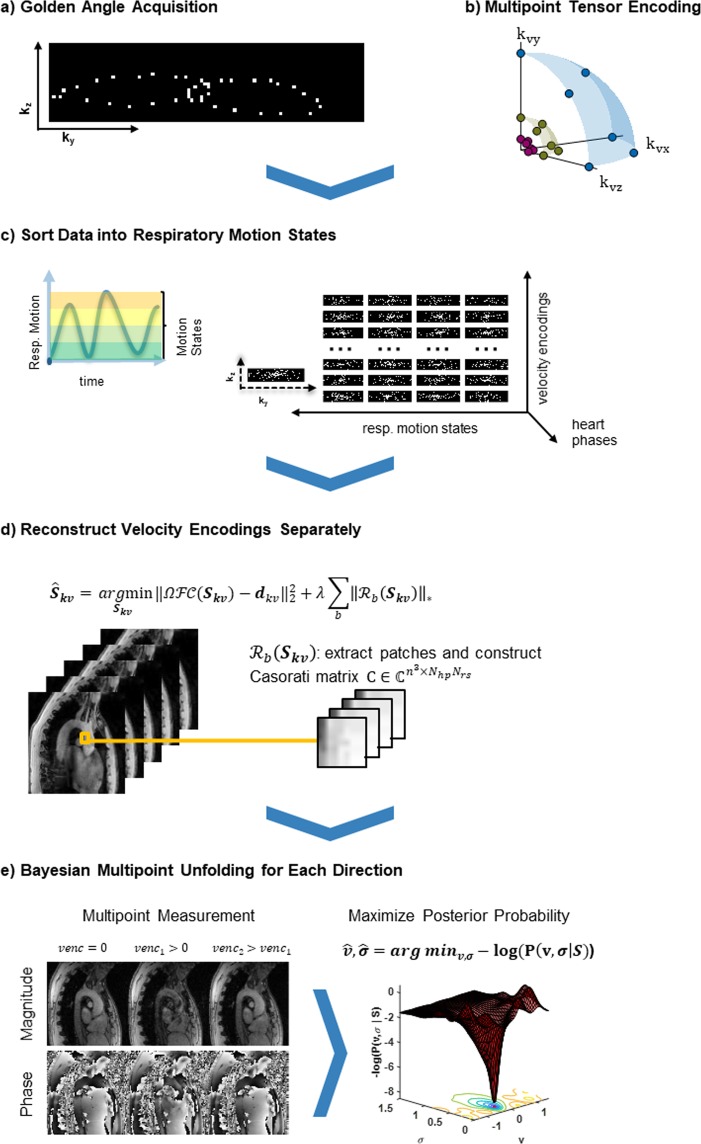
Illustration of *in-vivo* 5D Flow Tensor MRI: (**a**) K-space data are continuously acquired on a Cartesian golden angle trajectory during free breathing of the subject. (**b**) Velocities are encoded along six non-collinear directions with different velocity encodings VENC for improved accuracy of ISVD quantification over the desired range. (**c**) Each readout is assigned to a discrete respiratory motion state and cardiac phase, leading to undersampling patterns as required by compressed sensing reconstructions. (**d**) Images for each velocity encoding are reconstructed separately by exploiting correlations over cardiac and respiratory dimensions using a locally low-rank reconstruction. (**e**) For each direction, the measurements with different VENCs are combined using a Bayesian approach which selects the most likely values $\bar{{\boldsymbol{v}}}$ and ${\boldsymbol{\sigma }}$ given the signal model ${{\boldsymbol{S}}}_{kv}$ and the measured data ${{\boldsymbol{d}}}_{kv}$.

Figure 1 illustrates the 5D Flow Tensor MRI concept including data acquisition, multipoint encoding, data reconstruction and Bayesian processing. Data are sparsely sampled using a Cartesian golden angle trajectory and retrospectively sorted into discrete respiratory motion states and cardiac phases^19^. Each velocity encoding is reconstructed separately using a locally low-rank reconstruction approach. Velocities are encoded in six non-collinear directions using three velocity encodings per axis to cover the range of turbulence intensity and mean velocities for patients and healthy controls.

### Distributions of and sensitivity to intravoxel standard deviations

**Figure 2:**
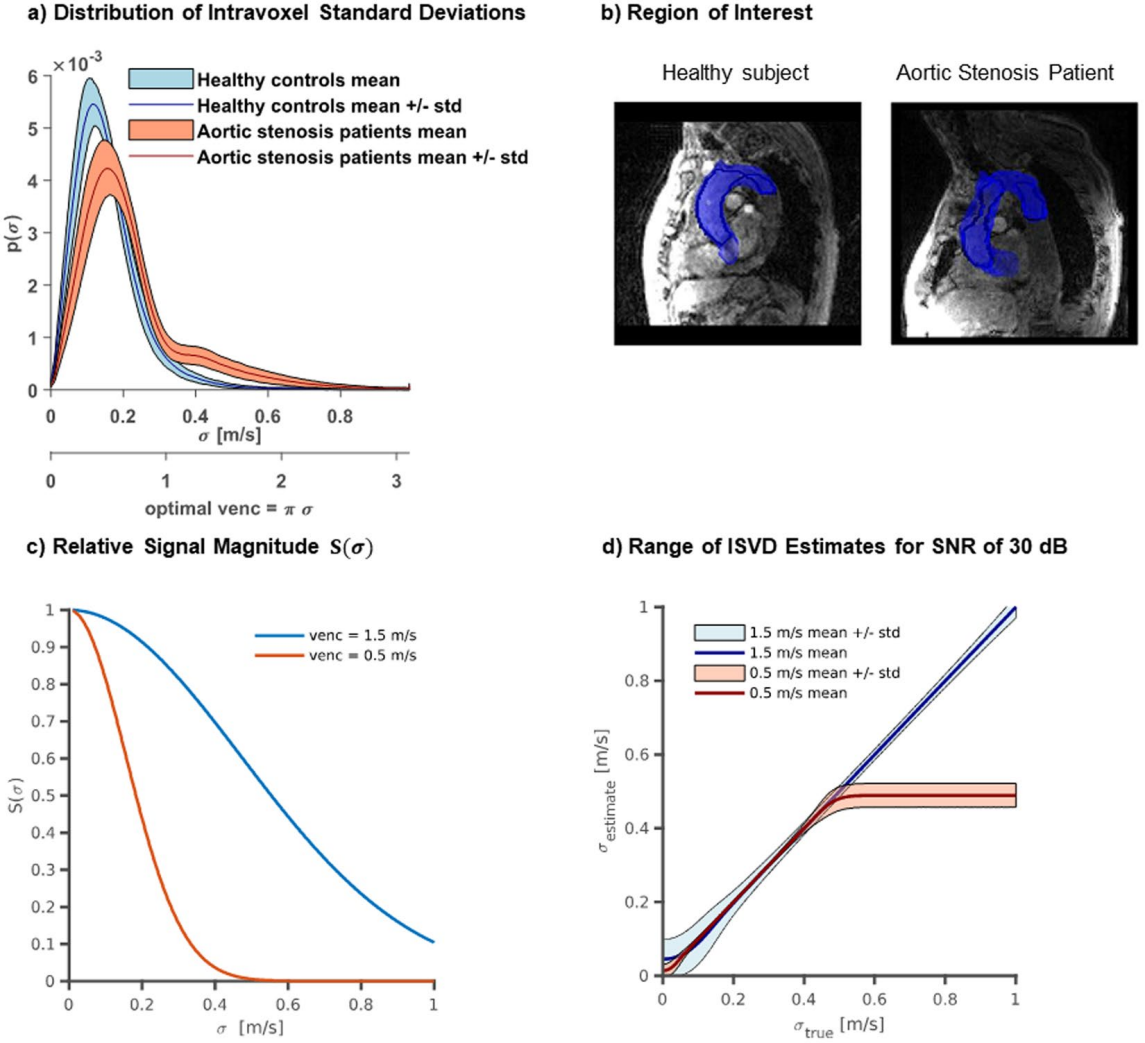
Exemplary distributions of IVSD in healthy and pathological aortae and illustration of IVSD encoding accuracy. (**a**) For healthy volunteers, IVSD is distributed mainly between 0 m/s and 0.3 m/s. For patients, a wider distribution can be observed with values of IVSD up to 0.8 m/s. (**b**) Examples of the region of interest for healthy controls and patients with aortic stenosis. (**c**) IVSD leads to a reduction in signal magnitude which depends on the encoding velocity VENC. The signal shows a high sensitivity to changes in IVSD within a limited range. For low values, the magnitude changes little, whereas for high values the signal vanishes completely. (**d**) Uncertainty of IVSD considering noisy data with an SNR of 30 dB. If ISVD is too high or too low, the IVSD estimates decrease in accuracy. Moreover, IVSDs for which the signal magnitude vanishes cannot be discerned and lead to a plateau in the plot.

To make an appropriate choice of the number and strength of velocity encodings per spatial axis, the distribution of velocity intravoxel standard deviations (IVSD)^8^ was compared based on retrospective 4D Flow MRI data of patients with moderate and severe aortic valve stenosis (N = 28) and healthy controls (N = 9) collected as part of a previous study^13^. As shown in Fig. 2a, ISVD reaches up to 0.8 m/s in patients, while peak ISVD values of 0.3 m/s are measured in healthy controls. Since the MR signal magnitude is non-linearly related to ISVD, velocity encodings per axis need to be distributed in a non-equidistant manner. As illustrated in Fig. 2c, a velocity encoding (VENC) of 0.5 m/s shows high sensitivity to IVSD in the healthy controls whereas a VENC of 1.50 m/s is optimal to probe IVSD in the aortic stenosis patients. Figure 2d illustrates the resulting uncertainty in IVSD quantification with noisy data. Using Monte-Carlo simulations, for each value of IVSD *σ*, 10^5^ samples with additive white Gaussian noise were generated and mean and standard deviation of the IVSD estimates *σ*_*est*_ were determined. In case *σ* is too high or too low, *σ*_*est*_ decreases in accuracy. Moreover, values of *σ* for which the signal magnitude vanishes cannot be discerned and lead to a plateau in the plot. As can be seen, an encoding velocity of 0.5 m/s, which would cover the range of IVSD in healthy aortae, cannot discern elevated values in patients. To ensure an accurate estimate of IVSD over the entire observed range, a distributed encoding scheme with 0.5 m/s, 1.5 m/s and 4.5 m/s is proposed. The first two values cover the range of turbulence, whereas the latter value prevents aliasing in the mean velocity field.

### Spatial resolution and Signal-to-Noise requirements

**Figure 3:**
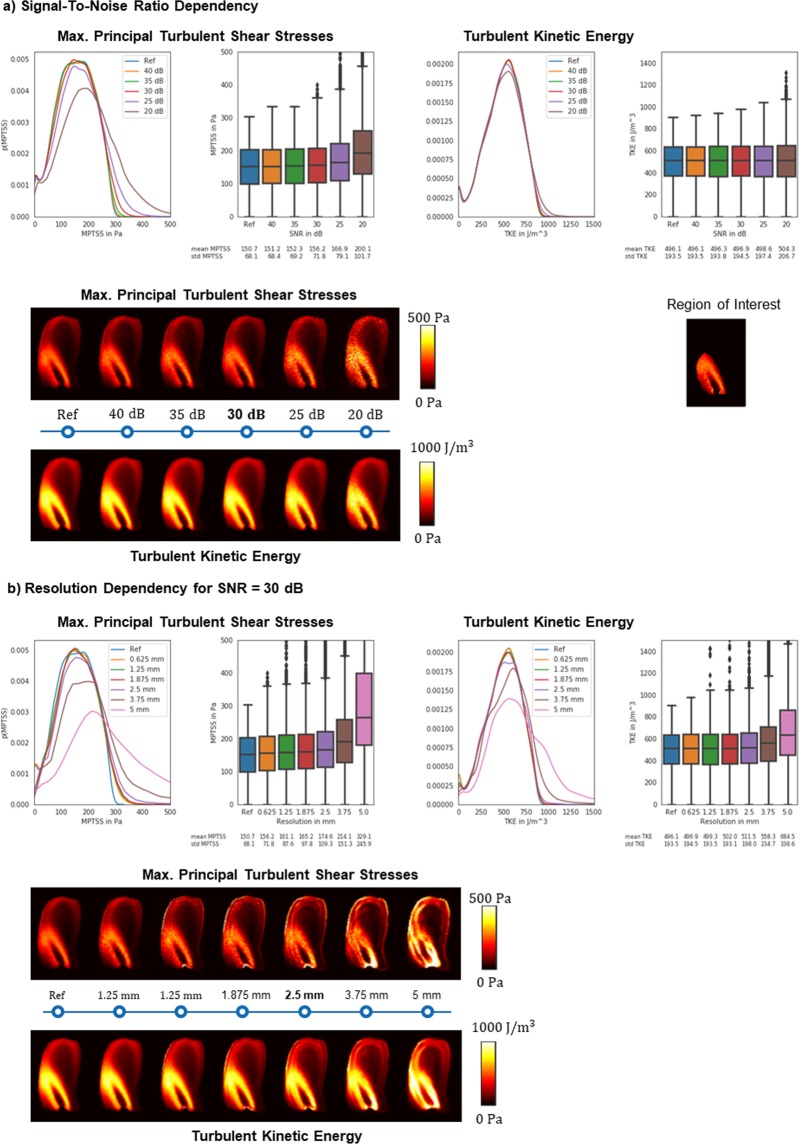
Impact of SNR and image resolution on quantification of TKE and MPTSS. (**a**) Decreasing SNR leads to an overestimation of TKE and MPTSS. At an SNR of 30 dB, as estimated for *in-vivo* experiments, this overestimation is relatively low. (**b**) Increasing voxel sizes lead to a skewed distribution of TKE and MPTSS. At a resolution of 2.5 mm, as used for *in-vivo* experiments, TKE is overestimated by 3.1% and MPTSS is overestimated by 15.9% on average.

The effect of different signal-to-noise ratios (SNR) and the impact of image resolution on TKE and maximum principal turbulent shear stress (MPTSS) quantification was assessed using data previously acquired with particle tracking velocimetry (PTV)^22^ as summarized in Fig. 3. For low SNR, an increase in mean values is observed for MPTSS. For TKE, the average mean values remain stable for low values of SNR (1.7% increase at 20 dB) while an increase in standard deviation is observed for decreasing SNR (e.g. 6.8% increase at 20 dB). At an SNR of 30 dB, as estimated for the *in-vivo* scans, MPTSS is overestimated by 3.6% on average whereas TKE values show no relevant increase in mean value (0.2%). Figure 3b shows the impact of different image resolutions for an SNR of 30 dB. Exemplary images show an increase of MPTSS and TKE at the jet core for increased voxel sizes. For large voxel sizes, the distribution of MPTSS values is skewed towards higher values with a corresponding increase in mean values and standard deviation. At a resolution of 2.5 mm, as used for the *in-vivo* exams, MPTSS are overestimated by 15.9% on average. TKE distributions are also skewed towards higher values for large voxel sizes with an overestimation of 3.1% at 2.5 mm.

Accuracy and precision of TKE and MPTSS quantification were simulated in a Monte-Carlo simulation with 40 repetitions. Mean and standard deviation over the repetitions are provided in Table 1 for varying SNR at the highest resolution and Table 2 for different resolutions at an SNR of 30 dB respectively. Table 1 shows an increase in the random error for decreasing SNR. However, the random error on mean and standard deviation of the value distribution remains below 1% for all metrics. Table 2 shows the effect of increasing voxel sizes for a fixed SNR of 30 dB. For increasing voxel sizes, a systematic overestimation can be observed for all metrics. Moreover, mean and standard deviation of MPTSS and TKE distributions show a higher random error for increased voxel sizes.

**Table 1:** Accuracy and precision for a resolution of 0.625 mm and varying SNR obtained in a Monte Carlo type experiment with 40 repetitions. For lower SNRs a bias towards higher values is observed and accuracy deteriorates.

SNR (Res = 0.625 mm)	TKE mean (mean ± std)	TKE std (mean ± std)	MPTSS mean (mean ± std)	MPTSS std (mean ± std)
40 dB	496.2 ± 0.0	193.6 ± 0.0	151.2 ± 0.0	68.4 ± 0.0
35 dB	496.4 ± 0.1	193.8 ± 0.1	152.4 ± 0.1	69.2 ± 0.1
30 dB	496.9 ± 0.1	194.6 ± 0.1	156.0 ± 0.1	71.6 ± 0.1
25 dB	498.6 ± 0.2	197.3 ± 0.2	167.0 ± 0.2	79.1 ± 0.2
20 dB	504.1 ± 0.4	206.1 ± 0.3	199.8 ± 0.4	101.5 ± 0.5

**Table 2:** Accuracy and precision for a SNR of 30 dB and varying resolution (voxel size) obtained in a Monte Carlo type experiment with 40 repetitions. Increasing voxel sizes lead to an overestimation of MPTSS and TKE. No clear trend can be observed for accuracy.

Resolution (SNR = 30 dB)	TKE mean (mean ± std)	TKE std (mean ± std)	MPTSS mean (mean ± std)	MPTSS std (mean ± std)
0.625 mm	496.9 ± 0.4	194.7 ± 0.1	156.0 ± 0.7	71.7 ± 0.2
1.25 mm	499.4 ± 0.9	193.5 ± 2.6	161.2 ± 1.2	88.8 ± 6.6
1.875 mm	502.3 ± 1.3	194.1 ± 4.4	165.4 ± 1.5	100.5 ± 9.7
2.5 mm	511.8 ± 1.4	198.9 ± 4.6	174.9 ± 1.6	110.7 ± 10.0
3.75 mm	558.7 ± 2.6	236.1 ± 10.3	214.5 ± 2.6	153.2 ± 16.4
5 mm	684.6 ± 2.2	338.0 ± 3.6	328.9 ± 1.4	245.4 ± 3.7

At 2.5 mm resolution and an SNR of 30 dB, TKE values show a mean of 511.8 ± 1.4 J/m^3^ and a standard deviation of 198.9 ± 4.6 J/m^3^ whereas MPTSS has a mean of 174.9 ± 1.6 Pa and a standard deviation of 110.7 ± 10.0 Pa.

### *In-Vivo* measurements

Flow in the aorta of two patients with a bioprosthetic aortic valve (65 yrs, female with a SJM Trifecta Aortic Valve TFGT-21A, 21 mm, and 80 years, female with an Edwards SAPIEN 3, 23 mm) and two healthy controls (26 yrs, female and 58 yrs, female) was acquired using the 5D Flow Tensor MRI approach on a clinical 1.5 T MRI system (Philips Healthcare, Best, The Netherlands) and a 5-channel receive array.

**Figure 4:**
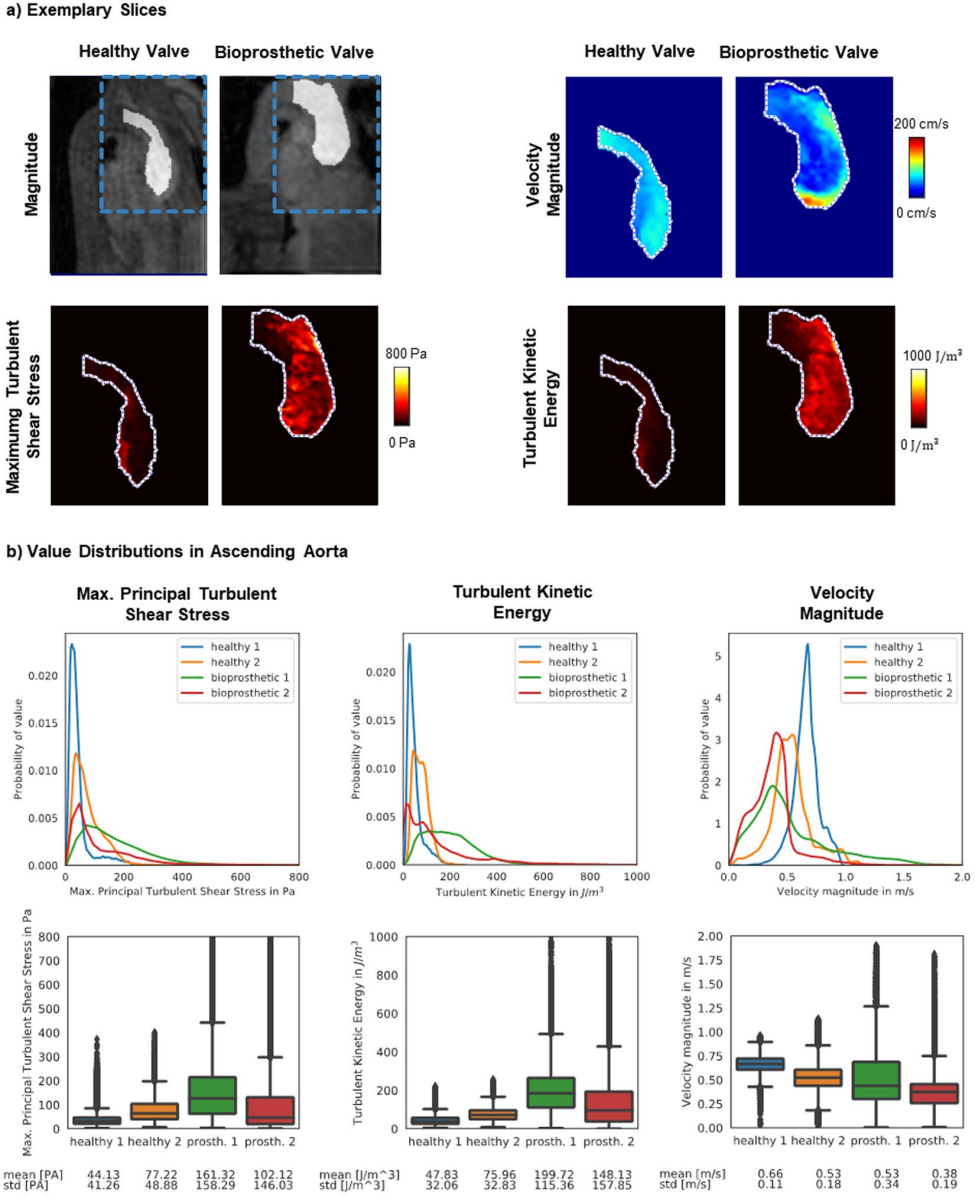
*In-vivo* assessment of turbulent flow through healthy and a bio-prosthetic heart valves. (**a**) Shows exemplary slices of a healthy and a bioprosthetic heart valve. The flow field shows uniform distribution of velocity magnitudes throughout the proximal aorta for the healthy valve whereas a jet with high velocities can be observed for the bio-prosthetic valve. MPTSS and TKE are elevated downstream of the bio-prosthetic valve. Visual assessment shows highest MPTSS and TKE near the vessel wall for the healthy valve and elevated values throughout the proximal aorta for the bio-prosthetic valve. (**b**) Shows value distributions for the different metrics, with *healthy 1* and *bioprosthetic 1* corresponding to the examples from (**a**). MPTSS and TKE are elevated for the bio-prosthetic heart valves. Velocities are on average lower for the bio-prosthetic heart valve but are distributed over a larger value range.

Figure 4a shows exemplary results in a single slice for a patient and a healthy control (patient 65 yrs, female, and volunteer 26 yrs, female). The highest values of TKE and MPTSS can be seen downstream of the bio-prosthetic valve in the patient. Figure 4b shows value distributions of velocity magnitudes, TKE, and MPTSS in the ascending aorta during systole. Increased values of TKE and MPTSS in the patients relative to the controls were found. (For TKE, patients: 199.7 ± 115.4 J/m^3^ and 148.1 ± 157.9 J/m^3^ vs. volunteers: 47.8 ± 32.1 J/m^3^ and 76.0 ± 32.8 J/m^3^, and for MPTSS, patients: 161.3 ± 158.3 Pa and 102.1 ± 146.0 Pa vs. volunteers: 44.1 ± 41.3 Pa and 77.2 ± 48.9 Pa). Mean velocities in the patients were 0.53 ± 0.34 m/s and 0.38 ± 0.19 m/s compared to 0.66 ± 0.11 m/s and 0.53 ± 0.18 m/s for the healthy controls.

## Discussion

This study has demonstrated *in-vivo* turbulent flow assessment using 5D Flow Tensor MRI in clinically feasible scan times for the first time. A multi-point encoding scheme was employed to probe the mean and fluctuating velocity components using non-collinear encoding directions, similar to concepts used in diffusion tensor imaging^23^. The approach permits, besides the assessment of time-resolved mean velocity vector fields, the quantification of Reynolds stresses and hence turbulent kinetic energy and turbulent shear stresses *in-vivo*.

Distributions of IVSD in the aortae of healthy subjects and patients with aortic valve disease were analyzed to choose velocity encodings (Fig. 2a,b). As illustrated in Fig. 2c,d, the choice of velocity encoding has considerable impact on the accuracy of IVSD quantification. This makes the choice of an appropriate encoding velocity crucial, when using a single encoding velocity per axis as in conventional 4D Flow MRI. To probe IVSD with an increased dynamic range, encoding of the RST was combined with a multipoint scheme^18^. In the present study, encoding velocities of 0.5 m/s, 1.5 m/s and 4.5 m/s were selected. As indicated in Fig. 2a, encoding velocities of 0.5 m/s and 1.5 m/s cover the expected range of IVSD. The additional velocity encoding at 4.5 m/s was used to avoid phase wraps in the reconstructed mean velocity fields. Of note, the particular choice of encoding velocities was made with respects to the range of observed IVSD, to prevent aliasing artifacts in the mean velocity fields, and to make echo times not too long. However, the encoding scheme yields further potential for optimization. In particular, the use of advanced phase unwrapping methods^24^ might allow to leave out the highest VENC.

Simulation of the MRI acquisition and encoding process revealed an overestimation of TKE and MPTSS for large voxel sizes. The overestimation amounted to about 3.1% for TKE and to approximately 15.9% for MPTSS at the given acquisition resolution of 2.5 mm and at an estimated SNR of 30 dB. The impact of image resolution can be related to the assumption of Gaussian intra-voxel velocity distributions in the derivation of turbulence, which is not fulfilled for coarse image resolutions as shown in previous studies^25^.

The impact of SNR on quantification of TKE and MPTSS was found to be relatively low compared to the impact of resolution. Starting at low SNR values below 25 dB, an overestimation of MPTSS was observed whereas TKE estimates were robust even at lower SNR values. SNR was estimated at ca. 30 dB in this study. In this range, noise played only a minor role in the assessment of TKE and MPTSS.

As shown in the Monte-Carlo simulation, the error in real-world experimental conditions is mostly due to a loss in accuracy for reduced image resolutions, whereas the random fluctuations for repeated experiments is comparatively low. However, increasing image resolution would lead to a decrease in SNR and noise would start to compromise the assessment of turbulent quantities. Therefore, rather than increasing acquisition resolution, efforts to mitigate the effect of large voxel sizes by e.g. data assimilation approaches^26^ are considered potential future options.

The feasibility of 5D Flow Tensor MRI to quantify distributions of MPTSS and TKE in patients with a bio-prosthetic valve relative to healthy controls has successfully been demonstrated. Distributions of TKE and MPTSS revealed distinct differences, while differences in mean velocity magnitudes were partly overlapping (Fig. 4b). In the healthy controls, the highest values of TKE and MPTSS were found near the vessel walls, which can be attributed to partial volume effects (there were also some differences between the two volunteers which can be related to the difference in age^27^). In contrast, flow downstream of the prosthetic valves showed highest values of MPTSS and TKE in the proximal aorta, reaching values of up to 500 Pa and 600 J/m^3^, respectively. MPTSS values were found to be below the threshold of elevated risk of red blood cell damage which was estimated between ca. 600 Pa^10^ and 800 Pa^28^. While mechanical heart valves have been associated with blood cell damage^12^, modern bio-prosthetic valves typically do not lead to complications^29^. An increase in shear stresses without reaching a critical level was therefore expected. It should, however, be noted that the implantation of bio-prosthetic valves is primarily indicated in the elderly population, while mechanical heart valves are preferred in younger patients. Accordingly, future work using 5D Flow Tensor MRI should include patients with mechanical heart valves to assess and compare TKE and MPTSS levels.

Of note, the fixed scan time of 10 minutes which was set for the *in-vivo* study was sufficient for all subjects examined in this study. However, in cases where patient geometry requires a much larger field of view, an increase in scan time might be required.

A limitation of the present study is that no ground truth data was available to assess the accuracy of the *in-vivo* scans. Accordingly, computer simulations were used to provide estimates of accuracy and precision. However, the simulations were based on PTV measurements with a resolution of 0.625 mm. Thus, the reference data were already subject to some discretization error and availability of higher resolution ground truth data might show an even higher overestimation of turbulence. Another practical drawback relates to the long data reconstruction times (ca. 1.5 h to 2 h on a workstation with two 14 Core Intel Xeon E5-2680 CPUs and 256 GB RAM) which implies that data evaluation can only be performed after the scan session. Currently ongoing work is addressing this inconvenience by using variational neural networks^30^ which have already been shown to perform compressed sensing reconstruction of standard 4D Flow MRI data in less than a minute^31^.

In conclusion, 5D Flow Tensor MRI provides comprehensive quantification of turbulent flow in clinically feasible scan times. Its ability to assess elevated TKE and MPTSS *in-vivo* has successfully been demonstrated. Efficient *in-vivo* turbulence quantification will contribute also to methods aiming at quantifying irreversible pressure loss downstream of heart valves and stenotic sections.

## Methods

### Measurement of reynolds stress tensor

In general, flow velocity vectors can be decomposed into a time-averaged mean vector $\bar{{\boldsymbol{v}}}$ and fluctuating components ${\boldsymbol{v}}\,{\boldsymbol{^{\prime} }}$ ^32^:

1 $${\boldsymbol{v}}=\bar{{\boldsymbol{v}}}+{\boldsymbol{v}}\,{\boldsymbol{^{\prime} }}.$$

In the one-dimensional case, assuming a Gaussian intra-voxel velocity distribution (IVSD) of variance *σ*^2^, the MR signal *S*(*k*_*v*_) reads^8^:

2 $$S({k}_{v})={S}_{0}{e}^{\frac{-{\sigma }^{2}{k}_{v}^{2}}{2}}{e}^{-i{k}_{v}\bar{v}}.$$

where ${k}_{v}=\gamma {\int }_{0}^{T}\,tG(t)dt=\frac{\pi }{VENC}$ denotes the first gradient moment of bipolar velocity encoding gradient *G* applied during time *T*.

The relationship described in Eq. 2 is non-linear and requires adjustment of the encoding strength according to the expected range of IVSD. By setting $\frac{{\delta }^{2}S({{\boldsymbol{k}}}_{{\boldsymbol{v}}})}{\delta {{\boldsymbol{\sigma }}}^{2}}=0,$ the optimal encoding strength for a given IVS D *σ* can be determined as ${k}_{v}=1/\sigma$ or $VENC=\pi \,\ast \,\sigma$.

The three-dimensional statistical description of velocity fluctuations ${\boldsymbol{v}}\,{\boldsymbol{^{\prime} }}$ includes variances and covariances as described by the RST (RST):

3 $${\boldsymbol{R}}=\rho [\begin{array}{ccc}\overline{{v}_{x}^{\text{'}}{v}_{x}^{\text{'}}} & \overline{{v}_{x}^{\text{'}}{v}_{y}^{\text{'}}} & \overline{{v}_{x}^{\text{'}}{v}_{z}^{\text{'}}}\\ \overline{{v}_{y}^{\text{'}}{v}_{x}^{\text{'}}} & \overline{{v}_{y}^{\text{'}}{v}_{y}^{\text{'}}} & \overline{{v}_{y}^{\text{'}}{v}_{z}^{\text{'}}}\\ \overline{{v}_{z}^{\text{'}}{v}_{x}^{\text{'}}} & \overline{{v}_{z}^{\text{'}}{v}_{y}^{\text{'}}} & \overline{{v}_{z}^{\text{'}}{v}_{z}^{\text{'}}}\end{array}]$$

with variances $\overline{{v}_{i}^{\text{'}}{v}_{i}^{\text{'}}}$, covariances $\overline{{v}_{i}^{\text{'}}{v}_{j}^{\text{'}}}$ and fluid density *ρ*. The magnitude of the complex-valued MR signal can be written as^14^:

4 $$|S({{\boldsymbol{k}}}_{{\boldsymbol{v}}})|=|{S}_{0}|{{\rm{e}}}^{-\frac{1}{2{\rm{\rho }}}{{{\boldsymbol{k}}}_{{\boldsymbol{v}}}}^{{\boldsymbol{T}}}{\boldsymbol{R}}{{\boldsymbol{k}}}_{{\boldsymbol{v}}}}$$

with $${{\boldsymbol{k}}}_{{\boldsymbol{v}}}={[{k}_{vx},{k}_{vy},{k}_{vz}]}^{T}$$.

Analogous to diffusion tensor imaging^23^, the RST can be determined by encoding along six non-collinear directions and solving a system of linear equations. For six measurements along six different velocity encodings and ${{\sigma }_{kv,i}}^{2}=\frac{2}{{|{{\boldsymbol{k}}}_{{\boldsymbol{v}},{\boldsymbol{i}}}|}^{2}}ln\frac{|S({{\boldsymbol{k}}}_{{\boldsymbol{v}}}=0)|}{|S({{\boldsymbol{k}}}_{{\boldsymbol{v}},{\boldsymbol{i}}})|}$, the following set of equations is obtained:

5 $$\begin{array}{rcl}(\begin{array}{c}{\sigma }_{{{\boldsymbol{k}}}_{v,1}}^{2}\\ \vdots \\ {\sigma }_{{{\boldsymbol{k}}}_{v,6}}^{2}\end{array}) & = & (\begin{array}{c}\frac{({{k}_{vx,1}}^{2}\,{{k}_{vy,1}}^{2}\,{{k}_{vz,1}}^{2}\,2{k}_{vx,1}{k}_{vy,1}\,2{k}_{vx,1}{k}_{vz,1}\,2{k}_{vy,1}{k}_{vz,1})}{{|{{\boldsymbol{k}}}_{{\boldsymbol{v}},1}|}^{2}}\\ \ldots \\ \ldots \\ \ldots \\ \frac{({{k}_{vx,6}}^{2}\,{{k}_{vy,6}}^{2}\,{{k}_{vz,6}}^{2}\,2{k}_{vx,6}{k}_{vy,6}\,2{k}_{vx,6}{k}_{vz,6}\,2{k}_{vy,6}{k}_{vz,6})}{{|{{\boldsymbol{k}}}_{{\boldsymbol{v}},6}|}^{2}}\end{array})\,(\begin{array}{c}\begin{array}{c}\overline{{v}_{x}^{\text{'}}{v}_{x}^{\text{'}}}\\ \overline{{v}_{y}^{\text{'}}{v}_{y}^{\text{'}}}\\ \overline{{v}_{z}^{\text{'}}{v}_{z}^{\text{'}}}\end{array}\\ \begin{array}{c}\overline{{v}_{x}^{\text{'}}{v}_{y}^{\text{'}}}\\ \overline{{v}_{x}^{\text{'}}{v}_{z}^{\text{'}}}\\ \overline{{v}_{y}^{\text{'}}{v}_{z}^{\text{'}}}\end{array}\end{array})\\  & = & {\boldsymbol{H}}(\begin{array}{c}\begin{array}{c}\overline{{v}_{x}^{\text{'}}{v}_{x}^{\text{'}}}\\ \overline{{v}_{y}^{\text{'}}{v}_{y}^{\text{'}}}\\ \overline{{v}_{z}^{\text{'}}{v}_{z}^{\text{'}}}\end{array}\\ \begin{array}{c}\overline{{v}_{x}^{\text{'}}{v}_{y}^{\text{'}}}\\ \overline{{v}_{x}^{\text{'}}{v}_{z}^{\text{'}}}\\ \overline{{v}_{y}^{\text{'}}{v}_{z}^{\text{'}}}\end{array}\end{array}).\end{array}$$

Accordingly, the elements of the RST can be calculated voxel-wise using the pseudoinverse:

6 $$(\begin{array}{c}\overline{{v}_{x}^{\text{'}}{v}_{x}^{\text{'}}}\\ \overline{{v}_{y}^{\text{'}}{v}_{y}^{\text{'}}}\\ \overline{{v}_{z}^{\text{'}}{v}_{z}^{\text{'}}}\\ \overline{{v}_{x}^{\text{'}}{v}_{y}^{\text{'}}}\\ \overline{{v}_{x}^{\text{'}}{v}_{z}^{\text{'}}}\\ \overline{{v}_{y}^{\text{'}}{v}_{z}^{\text{'}}}\end{array})={({{\boldsymbol{H}}}^{T}{\boldsymbol{H}})}^{-1}\,\,{\boldsymbol{H}}(\begin{array}{c}{\sigma }_{{k}_{v,1}}^{2}\\ \vdots \\ {\sigma }_{{k}_{v,6}}^{2}\end{array})\,.$$

In this study, matrix ***H*** was designed according to:

7 $${\boldsymbol{H}}=(\begin{array}{c}\begin{array}{ccc}1 & 0 & 0\\ 0 & 1 & 0\\ 0 & 0 & 1\end{array}\\ \begin{array}{ccc}1/\sqrt{2} & 1/\sqrt{2} & 0\\ 1/\sqrt{2} & 0 & 1/\sqrt{2}\\ 0 & 1/\sqrt{2} & 1/\sqrt{2}\end{array}\end{array}).$$

To mitigate the effect of non-linear encoding of the ISVD, a multipoint approach^18^ was used to probe the velocity field at different encoding strengths. Figure 1b illustrates the velocity encoding which encodes velocities in three orthogonal directions and their combinations along the diagonals with different encoding strengths. For each direction the different encoding velocities were combined with Bayesian multipoint unfolding^18^ as illustrated in Fig. 1e.

### Measurement of mean velocities

Redundant encoding schemes provide additional information for estimation of mean velocities^33^. Denoting the velocities encoded in *n* different directions by $\tilde{{\boldsymbol{V}}}={({{\boldsymbol{v}}}_{1},\ldots ,{{\boldsymbol{v}}}_{n})}^{{\boldsymbol{T}}}$ with corresponding velocity encodings $\{{{\boldsymbol{k}}}_{{\boldsymbol{v}},1},\,\ldots ,\,{{\boldsymbol{k}}}_{{\boldsymbol{v}},{\boldsymbol{n}}}\}$, the velocities in the Cartesian coordinate system ${{\boldsymbol{V}}}_{{\boldsymbol{cart}}}=diag({v}_{x},{v}_{y},{v}_{z})$ can be written as:

8 $$\tilde{{\boldsymbol{V}}}=(\begin{array}{c}{{\boldsymbol{k}}}_{{\boldsymbol{v}}1}/|{{\boldsymbol{k}}}_{{\boldsymbol{v}}1}|\\ \ldots \\ {{\boldsymbol{k}}}_{{\boldsymbol{vn}}}/|{{\boldsymbol{k}}}_{{\boldsymbol{vn}}}|\end{array}){{\boldsymbol{V}}}_{{\boldsymbol{cart}}}={\boldsymbol{A}}\,{{\boldsymbol{V}}}_{{\boldsymbol{cart}}}$$

A solution to this overdetermined system of linear equations is provided by the pseudo-inverse:

9 $$(\begin{array}{ccc}{v}_{x} & 0 & 0\\ 0 & {v}_{y} & 0\\ 0 & 0 & {v}_{z}\end{array})={{\boldsymbol{V}}}_{{\boldsymbol{cart}}}={({{\boldsymbol{A}}}^{T}{\boldsymbol{A}})}^{-1}\,\,{{\boldsymbol{A}}}^{T}\tilde{{\boldsymbol{V}}}.$$

### Value range of intravoxel standard deviations

Datasets previously obtained in 9 healthy volunteers and 28 patients with aortic valve stenosis^13^ were retrospectively analyzed to determine the range of IVSD occurring in the ascending aorta (Fig. 2b shows exemplary slices with the corresponding region of interest). The data were acquired and reconstructed with multipoint acquisition and Bayesian reconstruction^18^. Values of VENC were 4.50, 1.50, and 0.50 m/s for patients and 2.00, 0.67, and 0.40 m/s for the healthy control group.

The ascending aorta was segmented manually. To assess the distribution of the ISVD for the two groups, the relative probability $p(\sigma )$ of different values of IVSD in the segmented region was calculated for each subject and the mean and standard deviation of $p(\sigma )$ were determined over the patient cohort and the healthy control group respectively.

### Spatial resolution and Signal-to-Noise requirements

MRI acquisitions with varying SNR and image resolution were simulated based on flow through a 64% stenosis measured with particle tracking velocimetry (PTV). Details on acquisition and processing of the PTV data can be found in^25,34^. The dynamic and kinematic viscosity were 5.82 × 10^−3^ Pa and 4.85 × 10^−6^ m^2^/s, respectively, and the fluid density 1200 kg/m^3^. The velocity-to-noise ratio was determined to be larger than 10^3^. The PTV data were mapped onto a voxel size of $0.625\times 0.625\times 0.625\,{{\rm{mm}}}^{3}$.

Based on the PTV data, the MRI signal was calculated according to Eq. 4. Encoding velocities were 0.5, 1.5, and 4.5 m/s. To limit the effect of artifacts in the numerical study a median filter of size 3 was applied to the components of the RST.

To assess acquisition with different voxel sizes, the signal was transformed to k-space and sampled using a window function with a bandwidth inversely proportional to the desired downsampling rate. Complex-valued white Gaussian noise of different strength was added to the data to obtain the desired SNR

10 $${\rm{SNR}}=20\,\log (\frac{{\rm{Signal}}}{{\rm{SD}}({\rm{Noise}})})$$

which was calculated over all velocity encodings.

### *In-Vivo* measurements

*In-vivo* assessment of the RST was performed in two patients with bio-prosthetic aortic valves and two healthy controls on a 1.5 T MR system (Philips Healthcare, Best, The Netherlands). The study was approved by the Ethics Committee of the Canton of Zurich, Switzerland, and all subjects provided written informed consent.

Data were acquired using a cardiac- and respiratory-motion resolved Cartesian tiny golden angle acquisition scheme^35,36^ including the necessary velocity encodings for RST measurements. Acquisition and reconstruction of the data is illustrated in Fig. 1. During image reconstruction, data were sorted into four discrete respiratory motion bins. View sharing^37,38^ among respiratory motion states was used to ensure a minimum acceleration factor of 35 for each frame. Scan parameters were: voxel size of 2.5 mm × 2.5 mm × 2.5 mm, 25 cardiac phases, multipoint flow tensor encoding with VENCs of 0.5 m/s, 1.5 m/s, and 4.5 m/s, TE/TR = 3.9 ms/6.0 ms and scan duration of 10 minutes compared to 71 minutes for a fully sampled scan (which could increase by a factor of ca. 2 when using respiratory navigator gating).

Prior to reconstruction, noise pre-whitening was performed based on noise statistics from a separate scan acquired without radio-frequency excitation. Data for each velocity encoding strength and direction were reconstructed separately with BART^39^, enforcing a locally low-rank model^20,21^ along cardiac phases and respiratory motion states^19^. The signal estimate ${\hat{{\bf{S}}}}_{{\boldsymbol{kv}}}$ is thus obtained by iterative minimization of the cost term

11 $${\hat{{\boldsymbol{S}}}}_{{\boldsymbol{kv}}}=\mathop{\,arg\,{\rm{\min }}}\limits_{{{\boldsymbol{S}}}_{{\boldsymbol{kv}}}}\Vert \Omega  {\mathcal F} {\mathscr{C}}({{\boldsymbol{S}}}_{{\boldsymbol{kv}}})-{{\boldsymbol{d}}}_{kv}{\Vert }_{2}^{2}+\lambda \sum _{b}\Vert { {\mathcal R} }_{b}({{\boldsymbol{S}}}_{{\boldsymbol{kv}}}){\Vert }_{\ast }$$

with the undersampling operator Ω, Fourier transform ${\mathcal F}$, coil sensitivities ${\mathscr{C}}$ and k-space data ${{\boldsymbol{d}}}_{kv}$. The operator ${ {\mathcal R} }_{b}$ selects the *b*-th out of *N*_*b*_ blocks of size of size ${n}_{x}\times {n}_{y}\times {n}_{z}=\,22\times 22\times 22$ in the image from all ${N}_{hp}$ heart phase s and ${N}_{rs}$ respiratory motion states and transforms them into a Casorati matrix with dimensions ${n}_{x}{n}_{y}{n}_{z}\times {N}_{hp}{N}_{rs}$. The reconstruction favors solutions for which this local Casorati matrix is low-ranked by penalizing its nuclear norm. The regularization weight *λ* was set to $\lambda =0.005$. Both, block size and regularization weight were tuned for best agreement of magnitude images of the healthy control with a fully sampled reference measurement.

Following image reconstruction, only data in the expiratory motion state were considered for further processing. SNR in the measured data was determined using the pseudo-replica method^40^ with 40 repetitions averaged over the ascending aorta and over the velocity encodings. Of note, approximate linearity is assumed with locally low-rank reconstructions. Accordingly, using Gaussian distribution of noise, the pseudo-replica method was considered the best approximation for SNR assessment.

### Data analysis

Turbulent Kinetic Energy (TKE) in [J/m^3^] was calculated from the main diagonal of the RST as:

12 $$TKE=\frac{\rho }{2}(\overline{{v}_{x}^{\text{'}}{v}_{x}^{\text{'}}}+\overline{{v}_{y}^{\text{'}}{v}_{y}^{\text{'}}}+\overline{{v}_{z}^{\text{'}}{v}_{z}^{\text{'}}}).$$

Principal stress analysis was performed and the maximum principal turbulent shear stress (MPTSS) was calculated from the eigenvalues ${\delta }_{1} > {\delta }_{2} > {\delta }_{3}$ of the RST as:

13 $${\tau }_{max}=0.5({\delta }_{1}-{\delta }_{3})$$

assuming a density of blood of $\rho =1060\,kg/{m}^{3}$.

For quantitative evaluations of *in-vivo* data the ascending aorta was manually segmented using ITK-SNAP^41^ and for the simulated data, the flow jet in was masked.

### Statistical analysis

Value-distributions of TKE, MPTSS, and velocity magnitude were investigated using a Gaussian kernel density estimate^42,43^. Moreover, mean and standard deviations of the distributions were assessed.

Accuracy and precision of TKE and MPTSS quantification were assessed in a Monte-Carlo simulation with 40 repetitions and mean and standard deviation over the experiment repetitions were determined.

### Ethics approval

The study was approved by the Ethics Committee of the Canton of Zurich, Switzerland, and all subjects provided written informed consent. Imaging was performed at the Zurich University Hospital, Zurich, Switzerland. Anonymized data was analyzed at ETH Zurich with approval by the mentioned authority.

Written, informed consent was obtained before the experiment according to ethics approval and institutional guidelines.

## Data Availability

The data that support the findings of this study are available from the corresponding author upon reasonable request subject to restriction on use by the Ethics Committee of the Canton of Zurich. A demo script with exemplary data will be provided online upon acceptance of this manuscript.
